# Loss of 89K Pathogenicity Island in Epidemic *Streptococcus suis*, China

**DOI:** 10.3201/eid2206.152010

**Published:** 2016-06

**Authors:** Xiaolu Shi, Huiyan Ye, Jun Wang, Zhencui Li, Jingzhong Wang, Baoshan Chen, Ronghui Wen, Qinghua Hu, Youjun Feng

**Affiliations:** Zhejiang University School of Medicine, Hangzhou, China (X. Shi, H. Ye, J. Wang, Z. Li, Y. Feng);; Shenzhen Centre for Disease Control and Prevention, Shenzhen City, China (X. Shi, J. Wang, Q. Hu);; Guangxi University, Nanning City, China (H. Ye, J. Wang, B. Chen, R. Wen)

**Keywords:** Streptococcus suis, pathogenicity island, 89K, bacteria, China, pigs, pork, zoonoses, occupational exposure

**To the Editor:** *Streptococcus suis* serotype 2 (SS2) is a previously neglected, newly emerging human pathogen that causes occupational and opportunistic infections (*1**,**2*). Outbreaks of fatal human SS2 infections in China, featuring streptococcal toxic shock syndrome, in 2005 seriously challenged global public health (*3*–*5*). The epidemic strain is unusual in that it contains a unique 89-kb (89K) pathogenicity island (PAI) (*3**,**6**,**7*). We observed the loss of genes from the 89K PAI in sporadic cases in southern China in 2007, implying the dynamic evolution of this PAI (*8*). Therefore, 89K PAI might be able to be used to monitor prevalent strains of *S. suis* in China (*8*). 

We report 10 recurrent cases of human *S. suis* infections during 2008–2015 in southern China. Most of the hospitalized patients were male workers in close contact with pigs, pork products, or both. These patients typically exhibited clinical syndromes of meningitis, including headache, coma, vomiting, and fever. The bacterial strains acquired from humans were as follows: 2 isolates in 2008 (Stre08001 and Stre08002), 2 in 2009 (Stre09001 and Stre09002), 2 in 2011 (Stre11001 and Stre11002), 3 in 2013 (Stre13002, Stre13003, and Stre13004), and 1 in 2015 (Stre15001). Microbial and molecular assays proved that these clinical isolates were *S. suis* (Technical Appendix Figures 1, 2). Multiplex PCR–based molecular determination (*16S rDNA*, *mrp*, *epf*, and *cps-2j*) suggested that all strains except Stre13002 were SS2 (Technical Appendix Figure 2) (*3*). To determine whether these clinical isolates derived from the same Chinese epidemic clone 05ZYH33, we sequenced an array of virulence factor–encoding genes, as well as the *16S rDNA* genes. Phylogenetic trees indicated that all 10 clinical strains are classified into the same subclade as that of the strain 05ZYH33 (Technical Appendix Figure 2).

Subsequent analyses by pulsed-field gel electrophoresis revealed that genotypes are diverse among these clinical strains, which can be roughly divided into 6 groups (Technical Appendix Figure 3). Given that the Sao surface antigen protein possesses 3 allelic variants (Sao-L [670 aa], Sao-M [580 aa], and Sao-S [489/490 aa]) (9), we thus assayed it with these clinical strains. Unexpectedly, we found 2 more new allelic variants, referred to as Sao-L1 (640 aa) and Sao-L2 (611 aa). Except for the strain BM407, which is a Chinese epidemic SS2 encoding Sao-L1, a version 30 residues shorter than Sao-L, 8 of the 10 clinical *S. suis* isolates consistently had the same new form of Sao protein, Sao-L2 (611 aa) (Technical Appendix Figure 4).

Because 89K PAI is in dynamic evolution, determining whether it remains present in clinical strains is of interest. As previously designed (Technical Appendix Table 1), a specific pair of boundary primers (1/6) was applied for PCR-based detection of the 89K PAI (Figure, panel A). In principle, the PCR-positive result suggests the absence of 89K PAI, whereas the PCR-negative result indicates the presence of 89K PAI (*6**,**8*). Unlike the epidemic strain 05ZYH33 that has the 89K PAI, 9 of the 10 clinical strains examined (Stre08001, Stre08002, Stre09001, Stre09002, Stre11001, Stre11002, Stre13002, Stre13003, and Stre13004) were unexpectedly found to be PCR positive for the unique 1/6 DNA fragment with expected size of ≈1.5 kb (Figure, panel B). This finding indicates that the 89K PAI is lost in these 9 clinical strains. We saw similar scenarios in the subsequent PCRs for other inner genes/DNA fragments (943 and 944 [*10*]; 1/2, 3/4, and 5/6 [*8*]) inside of 89K PAI (Figure, panel C). Further DNA sequencing of the 1/6 PCR product showed that it matches well with the 2 boundary regions neighboring the 89K PAI, validating the loss of 89K PAI in these 9 clinical isolates (Figure, panel D). In contrast, the strain Stre15001 behaved similarly to that of the 05ZYH33 containing the 89K PAI, in that both are PCR positive for the 4 amplicons of 1/2, 5/6, 943, and 944 but PCR negative for the 1/6 amplicon (Figure, panels B–D). The only minor difference between strains Stre15001 and 05ZYH33 lay in the 3/4 amplicon (Figure, panel C). Clearly the 3/4 DNA fragment is present in the 89K PAI from strain 05ZYH33 but not in the counterpart of the strain Stre15001 (Figure, panel C); that is, strain Stre15001 carries a variant of 89K PAI lacking (at least part of, if not all) the 3/4 DNA fragment. In terms of 89K PAI (and pulsed-field gel electrophoresis/Sao protein), we propose that a heterogeneous SS2 population is circulating in China. Also, we observe that the differentiation of bacterial virulence is related to the clinical strains using the infection model of Balb/c mice (Technical Appendix Figure 5).

**Figure:**
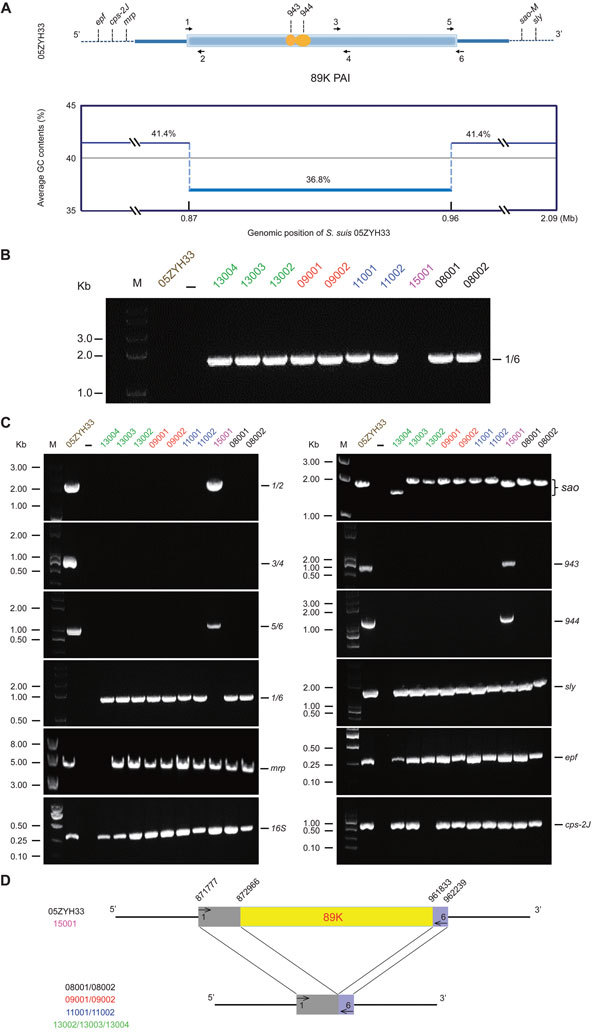
Molecular dissection for the 89K pathogenic island (PAI) in epidemic *Streptococcus suis* and the relevant virulence factors, China. A) Scheme for the 89K PAI and the relevant virulence factors on the bacterial chromosome of 05ZYH33, a representative isolate from the 2005 China outbreak in humans. 89K PAI is characterized with a light-blue column in which the important 2-component system-encoding genes (*943* and *944*) are highlighted in orange. Dashed lines indicate the genomic sequences outside of 89K. Black arrows indicate the 3 pairs of specific primers (refers to 1, 2, 3…6) used for the 89K assay. Five other genes outside of 89K (*epf*, *cps-2J*, *mrp*, *sao-M*, and *sly*) are labeled in black. The average GC value (guanine-cytosine content) of 89K is 36.8%, dramatically lower than the 41.1% of the whole genome of 05ZYH33. B) Probing the presence of 89K PAI in human *S. suis* isolates. The pair of specific primers 1 plus 6 is applied to determine whether the 89K PAI is present in the bacterial isolates. Determination criteria can be described as follows: PCR-negative results imply the existence of 89K; successful amplification of a DNA fragment in ≈1.5 kb indicates the absence of 89K. Except that Stre15001, a human *S. suis* serotype 2 isolate from the meningitis patient in 2015, contain the 89K PAI, all other 9 isolates are unexpectedly found not to carry 89K, which direct DNA sequencing further verified. C) Molecular characteristics of human *S. suis* isolates collected during 2008–2015 in Shenzhen City. 05ZYH33 here functions as the positive control; –, negative control. All the primers used here are listed in Technical Appendix Table 1), and the relevant PCR products were separated in 1.0% agarose gel. In total, the 12 genes/DNA fragments of interest were *1/2*, *3/4*, *5/6*, *1/6*, *mrp*, *16S*, *sao*, *943*, *944*, *sly*, *epf*, and *cps-2J*, respectively. D) Scheme for the loss of the 89K PAI among the Chinese *S. suis* strains. The numbers 1 and 6 represent a pair of specific primers designed for the detection of 89K PAI. Yellow indicates the 89K PAI. The 10 newly isolated strains are listed as 08001, 08002, 09001, 09002, 11001, 11002, 13002, 13003, 13004, and 15001.

In summary, the loss of 89K PAI might highlight the emergence of an epidemic SS2 population. This population appears to have genetic heterogeneity that is undergoing evolution in an adaption to some selection pressure from the environment, host restriction, or both.

Technical Appendix Primers carried for PCR-based molecular identification of epidemic *Streptococcus suis*, China; microbial characterization of the representative isolate of human *S. suis*; 16S rDNA-based phylogeny of the newly isolated *S. suis* strains; pulsed-field gel electrophoresis analyses for the newly isolated Chinese *S. suis* strains; heterogeneity of Sao surface protein from the *S. suis* populations; and virulence differentiation the Chinese *S. suis* population in the mice-based infection model.
