# Supplementary material for: Loss of 89K Pathogenicity Island in Epidemic Streptococcus suis, China
Source: Emerg Infect Dis. 2016 Jun;22(6):1126–7. doi: 10.3201/eid2206.152010 (PMC4880084; doi:10.3201/eid2206.152010)
Supplement: Technical Appendix — Primers carried for PCR-based molecular identification of epidemic Streptococcus suis, China; microbial characterization of the representative isolate of human S. suis; 16S rDNA-based phylogeny of the newly isolated S. suis strains; pulsed-field gel electrophoresis analyses for the newly isolated Chinese S. suis strains; heterogeneity of Sao surface protein from the S. suis populations; and virulence differentiation the Chinese S. suis population in the mice-based infection model. [file 15-2010-Techapp-s1.pdf]

Article DOI: http://dx.doi.org/10.32032/eid2206.152010

Loss of 89K Pathogenicity Island in Epidemic *Streptococcus suis*, China

Technical Appendix

Technical Appendix Table 1. Primers carried for PCR-based molecular identification o4f epidemic *Streptococcus suis*, China

Primer	Primer sequence	Reference	Target gene (kb)
1	5′-CACGCATCTCGTAGAGTTTGAC-3′	(*1*,*2*)	1/2 (≈2.1)
2	5′-AGATTGCGAGGCTTTTAGATTG-3′		
3	5′-TCGCCACTATGGTATCTGCTTA-3′	(*1*,*2*)	3/4 (≈0.7)
4	5′-GATTGTGGACCATGCTGTTTAG-3′		
5	5′-ATAAATAGCCCCATCCTCATCA-3′	(*1*,*2*)	5/6 (≈0.8)
6	5′-GCGTAGCTGCTTAGTGCTACAA-3′		1/6 (≈1.5)
943-F	5′-TTGAAAATTTTATTAATAGATG ATC-3′	(*2*)	‘943’(≈0.6)
943-R	5′-TTACTTAATATATCCTAACTTCTG-3′		
944-F	5′-TTGTTTTTTCAA AAGTTACAGAC-3′	(*2*)	‘944’ (≈1.2)
944-R	5′-TTATCTATTAATAAAATTTTCAATTGCC-3′		
16S-F	5′-CAGTATTTACCGCATGGTAGATAT-3′	(*2*)	*16S rDNA* (≈0.3)
16S-R	5′-GTAAGATACCGTCAAGTGAGAA-3′		
mrp-F	5′-GGTATACCTTGCTGGTACCGTTC-3′	(*2*)	*mrp* (≈0.6)
mrp-R	5′-AGTCTCTACAGCTGTAGCTGG-3′		
epf-F	5′-ACAAAGGCGTAGGTTCAATC-3′	(*2*)	*epf* (≈0.3)
epf-R	5′-CGGCATCAAGAATGTCTTTG-3′		
sao-F	5′-ATGAATACTAAGAAATGGAG-3′	(*3*)	*sao-M* (≈1.8)
sao-R	5′-TTATAATTTACGTTTACGTGT-3′		
2J-F	5′-TGATAGTGATTTGTCGGGAGGG-3′	(*2*,*4*)	*cps-2J* (≈0.5)
2J-R	5′-GAGTATCTAAAGAATGCCTATTG-3′		
sly-F	5′-GCAGATTCCAAACAAGAT-3′	(*2*)	*Suilysin* (≈1.4)
sly-R	5′-CTCTATCACCTCATCCGC-3′		

*The PCR experiments were conducted according to the primer combinations below (1&2, 3&4, 5&6, and 1&6).

References

1. Chen C, Tang J, Dong W, Wang C, Feng Y, Wang J, et al. A glimpse of streptococcal toxic shock syndrome from comparative genomics of *S. suis 2* Chinese isolates. PLoS One. 2007;2:e315. PubMed http://dx.doi.org/10.1371/journal.pone.0000315

2. Feng Y, Shi X, Zhang H, Zhang S, Ma Y, Zheng B, et al. Recurrence of human *Streptococcus suis* infections in 2007: three cases of meningitis and implications that heterogeneous *S. suis* 2 circulates in China. Zoonoses Public Health. 2009;56:506–14. PubMed http://dx.doi.org/10.1111/j.1863-2378.2008.01225.x

3. Feng Y, Zheng F, Pan X, Sun W, Wang C, Dong Y, et al. Existence and characterization of allelic variants of Sao, a newly identified surface protein from *Streptococcus suis*. FEMS Microbiol Lett. 2007;275:80–8. PubMed http://dx.doi.org/10.1111/j.1574-6968.2007.00859.x

4. Tang J, Wang C, Feng Y, Yang W, Song H, Chen Z, et al. Streptococcal toxic shock syndrome caused by *Streptococcus suis* serotype 2. PLoS Med. 2006;3:e151 Erratum in: PLoS Med. 2006;3:e377. PubMed http://dx.doi.org/10.1371/journal.pmed.0030151

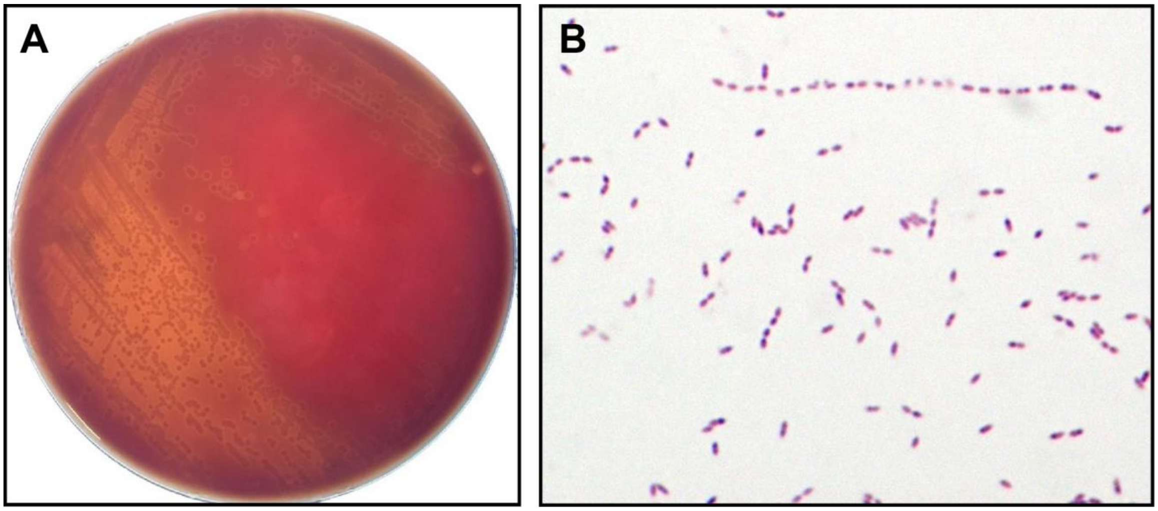

Technical Appendix Figure 1. Microbial characterization of the representative isolate of human *Streptococcus suis*, China. A) Colony phenotype of the isolated *S. suis* growing on the THB agar plate supplementing 5% sheep blood Yellow indicates the hemolytic activity. B) Light microscopic analyses of the *S. suis* cultures after Gram staining.

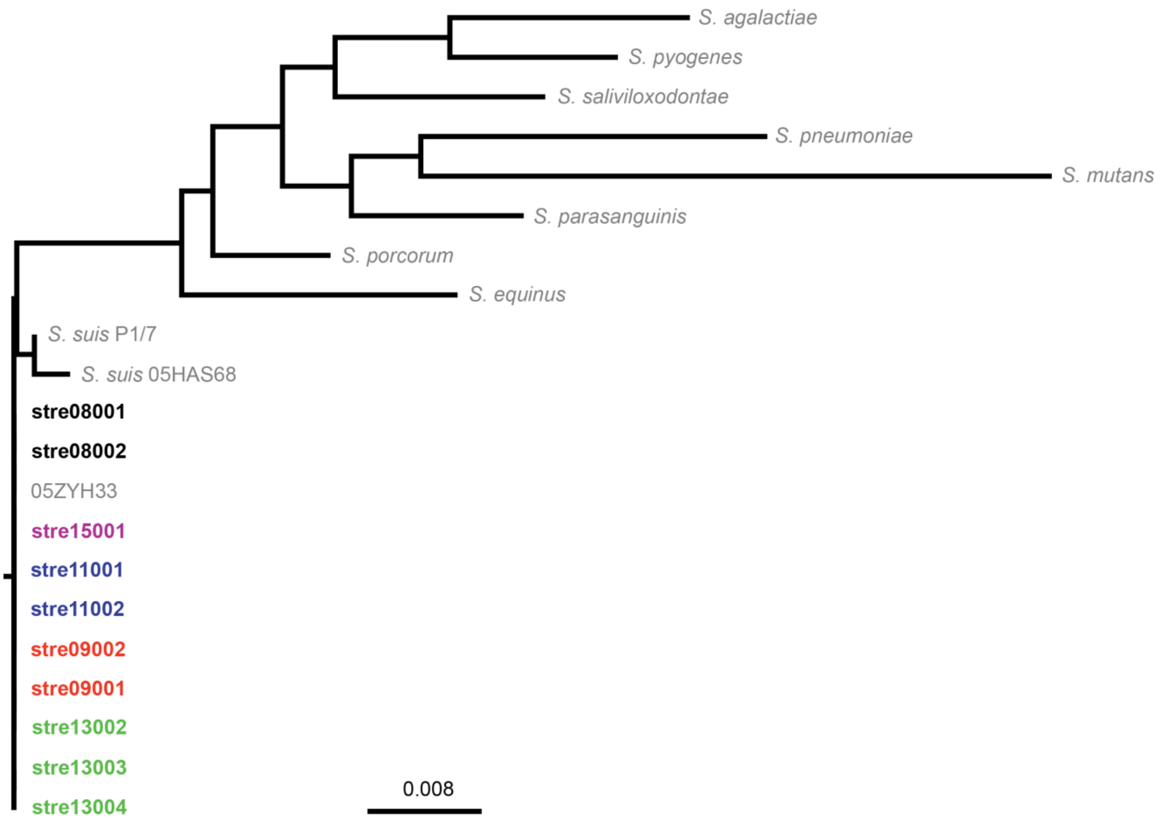

Technical Appendix Figure 2. 16S rDNA-based phylogeny of the newly isolated *Streptococcus suis* strains. The phylogenetic tree was constructed by using the program of ClustalW2 (http://www.ebi.ac.uk/Tools/msa/clustalw2), and the final output was given with the software of FigTree (http://tree.bio.ed.ac.uk/software/figtree). The international reference strain is *S. suis* 2 is P1/7, and the 2 known Chinese *S. suis* serotype 2 strains included 05ZYH33, an epidemic Chinese virulent strain and an avirulent strain 05HAS68. The 10 strains identified from Shenzhen City are in bold and color.

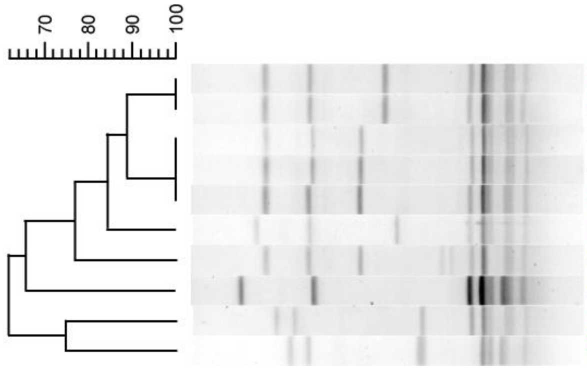

Isolates	City	Year	Presence of 89K
Stre13002	Shenzhen	2013	No
Stre13003	Shenzhen	2013	No
Stre08001	Shenzhen	2008	No
Stre08002	Shenzhen	2008	No
Stre09002	Shenzhen	2009	No
Stre13004	Shenzhen	2013	No
Stre11001	Shenzhen	2011	No
Stre11002	Shenzhen	2011	No
Stre09001	Shenzhen	2009	No
Stre15001	Shenzhen	2015	Yes

Technical Appendix Figure 3. PFGE analyses for the newly isolated Chinese *Streptococcus suis* strains. The genomic DNA of 10 bacterial species were isolated and digested with SmaI in gel. Clustering of PFGE patterns was conducted by an unweighted group with arithmetic averaging. The dentrogram of PFGE profiles was drawn using BioNumerics software 3.0 (Applied Maths, Austin, TX, USA). 89K is an abbreviation for a 89-kb pathogenicity island. PFGE, pulsed-field gel electrophoresis.

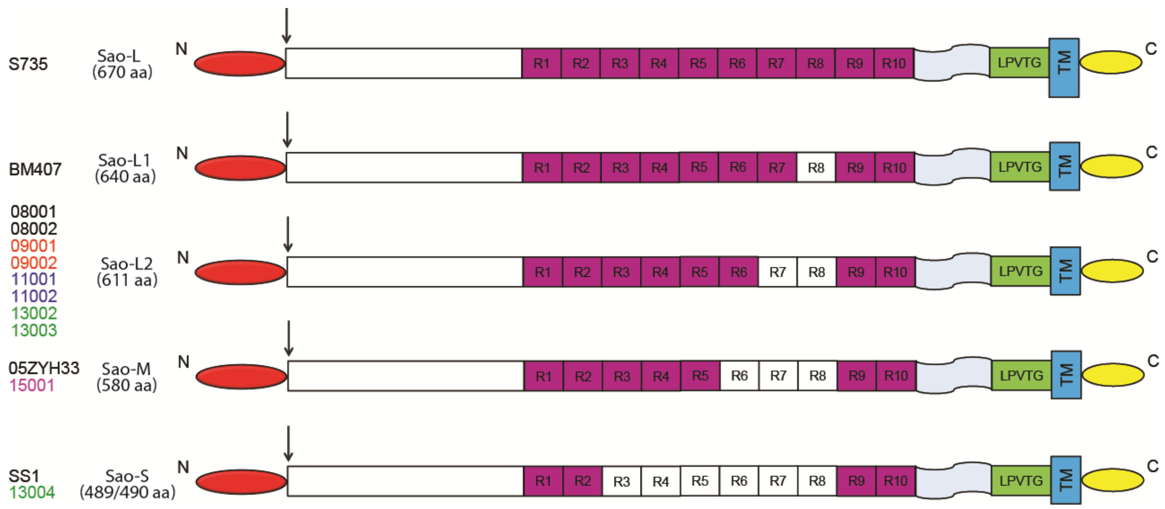

Technical Appendix Figure 4. Heterogeneity of Sao surface protein from the *Streptococcus suis* populations. Linear diagram for the 5 types of Sao proteins is given. The vertical arrow indicates the predicted cleavage site by signal peptidase. The ellipses in red and yellow depict the hydrophobic region at the N-terminus and the charged amino acids of the C-terminal tail. TM indicates the putative transmembrane region at the C-terminus. Curved grey boxes indicate the possible cell wall-associated regions. White boxes indicate deletion of repeated regions. Five types of Sao proteins were attributed to *S. suis* population, adding 2 more new forms (Sao-L1 and Sao-L2) to the former scenarios (Sao-L, Sao-M, and Sao-S). Sao-L: SaoO protein of 670 aa long; Sao-L1: Sao protein of 640 aa long; Sao-L2: Sao protein of 611 aa long; Sao-M: Sao protein of 580 aa long; Sao-S: Sao protein of 489/490 aa long. Multiple-sequence alignment of Sao protein suggested that the repeat regions are present in the C-terminus, but the number of repeat regions varies. R1, R2, R3 . . . R10: Repeated region1, 2, 3 . . . 10 in the C-terminus of Sao. S735: Holland isolate of *S. suis* 2 (Sao-L); BM407: A Chinese virulent strain of *S. suis* 2; SS1: Holland strain 5428 of *S. suis* 1 (Sao-S); 05ZYH33: an epidemic strain isolated from human *S. suis* 2 outbreak in China, 2005 (Sao-M). All the other *S. suis* strains reported here are numbered/indicated in color.

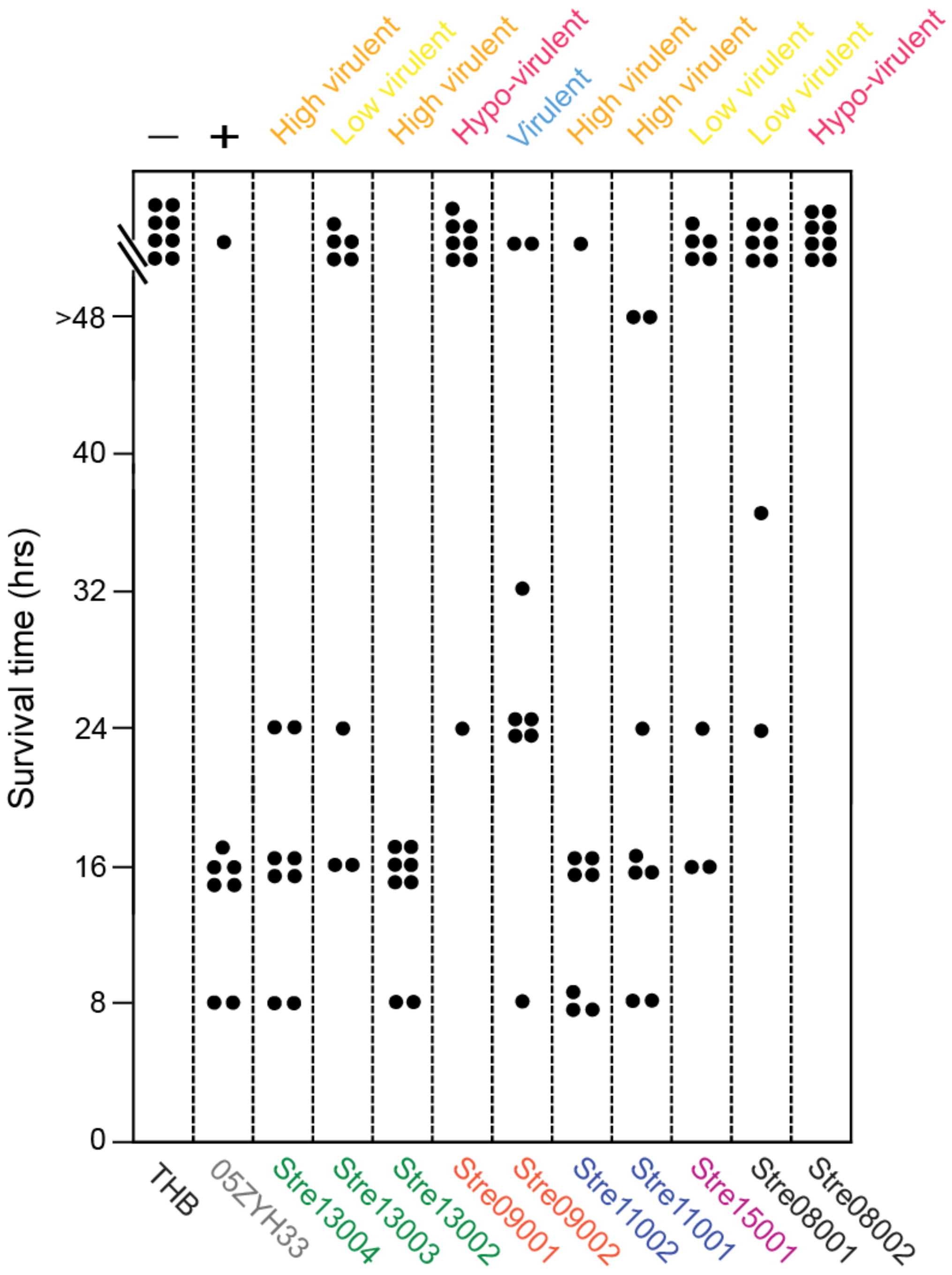

Technical Appendix Figure 5. Virulence differentiation the Chinese *Streptococcus suis* population in the mice-based infection model. In total, 12 groups of Balb/c mice (8 mice each group) were subjected to intraperitoneal (I.P.) injection with *S. suis* strains at a dose of 10^9CFU/mouse. The wild type/virulent strain of *S. suis*, 05ZYH33, acts as positive control (+); THB indicates blank control (--). The 10 strains of clinically isolated *S. suis* included Stre08001, Stre08002, Stre09001, Stre09002, Stre11001, Stre11002, Stre13002, Stre13003, Stre13004, and Stre15001, respectively. Survival time (hrs) of individual mouse is monitored during the entire period of infection. Here, the virulence of these newly collected clinical *S. suis*

isolates is differentiated into 4 groups: high virulent, virulent, low virulent, and hypovirulent (nonvirulent). The criteria for bacterial virulence differentiation are as follows: 1) high virulence for 7--8 deaths among the 8 infected mice (7/8--8/8); 2) virulence for no less than 6 deaths among the 8 challenged mice (6/8); 3) low virulence, 2–3 deaths among the 8 mice after -infection (2/8--3/8), and 4) hypovirulence (nonvirulence) decodes no more than 1 death among the 8 mice after the challenge (0/8--1/8).
